# Author Correction: Mutation bias reflects natural selection in *Arabidopsis thaliana*

**DOI:** 10.1038/s41586-023-06387-9

**Published:** 2023-07-26

**Authors:** J. Grey Monroe, Thanvi Srikant, Pablo Carbonell-Bejerano, Claude Becker, Mariele Lensink, Moises Exposito-Alonso, Marie Klein, Julia Hildebrandt, Manuela Neumann, Daniel Kliebenstein, Mao-Lun Weng, Eric Imbert, Jon Ågren, Matthew T. Rutter, Charles B. Fenster, Detlef Weigel

**Affiliations:** 1 Department of Molecular Biology, Max Planck Institute for Biology Tübingen, Tübingen, Germany; 2 Department of Plant Sciences, University of California Davis, Davis, CA USA; 3 Department of Plant Biology, Carnegie Institution for Science, Stanford, CA USA; 4 Department of Biology, Stanford University, Stanford, CA USA; 5 Department of Biology, Westfield State University, Westfield, MA USA; 6 ISEM, University of Montpellier, Montpellier, France; 7 Department of Ecology and Genetics, EBC, Uppsala University, Uppsala, Sweden; 8 Department of Biology, College of Charleston, Charleston, SC USA; 9 Oak Lake Field Station, South Dakota State University, Brookings, SD USA; 10 Present Address: Faculty of Biology, Ludwig Maximilian University, Martinsried, Germany

**Keywords:** Molecular evolution, Genetic variation, Epigenomics, Mutation

Correction to: *Nature* 10.1038/s41586-021-04269-6 Published online 12 January 2022

The second sentence in the Methods section “Identification of de novo somatic mutations in a resequencing dataset of *A. thaliana* leaves” has been corrected from “Raw fastq files were downloaded from NCBI and mapped to the TAIR10 reference genome using bwa-mem...” to “Raw fastq files were downloaded from NCBI and forward reads were mapped twice to the TAIR10 reference genome using bwa-mem (bwa mem ${sample}_**R1**.fastq.gz ${sample}_**R1**.fastq.gz)...”. No other text, figures or results have been altered.

In our study^1^, we tested whether trends of lower mutation rates in gene bodies and in essential genes observed in our initial set of sequence data were also observable in other datasets. To this end, we turned to several additional independent datasets, including published deep sequencing reads from 64 *A. thaliana* leaves^2^. We have since found code in the pipeline used only for this dataset that led to forward reads being mapped twice to the genome. This led to a large number of singletons being inadvertently called as potential non-singleton variants, potentially increasing the fraction of sequencing errors in this dataset (cf. Extended Data Fig. 4c in our study^1^). However, post hoc analyses of this specific subset of data analyzed in our study^1^ confirmed that the reported distribution of those variants is inconsistent with being explained by sequencing errors alone. A detailed discussion is found in Supplementary Note 4 in ref. ^3^.

We thank colleagues whose comments led us to revisit our code and apologize for the confusion this may have caused.
