# Supplementary material for: Mutation bias reflects natural selection in Arabidopsis thaliana
Source: Nature. 2022 Jan 12;602(7895):101–5. doi: 10.1038/s41586-021-04269-6 (PMC8810380; doi:10.1038/s41586-021-04269-6)
Supplement: Supplementary file 1 — Reporting Summary [file 41586_2021_4269_MOESM1_ESM.pdf]

nature research

Corresponding author(s): Detlef Weigel; Grey Monroe

Last updated by author(s): Nov 15, 2021

Reporting Summary

Nature Research wishes to improve the reproducibility of the work that we publish. This form provides structure for consistency and transparency in reporting. For further information on Nature Research policies, see our Editorial Policies and the Editorial Policy Checklist.

Statistics

For all statistical analyses, confirm that the following items are present in the figure legend, table legend, main text, or Methods section.

n/a	Confirmed	
☐	☒	The exact sample size (*n*) for each experimental group/condition, given as a discrete number and unit of measurement
☐	☒	A statement on whether measurements were taken from distinct samples or whether the same sample was measured repeatedly
☐	☒	The statistical test(s) used AND whether they are one- or two-sided<br>*Only common tests should be described solely by name; describe more complex techniques in the Methods section.*
☐	☒	A description of all covariates tested
☐	☒	A description of any assumptions or corrections, such as tests of normality and adjustment for multiple comparisons
☐	☒	A full description of the statistical parameters including central tendency (e.g. means) or other basic estimates (e.g. regression coefficient) AND variation (e.g. standard deviation) or associated estimates of uncertainty (e.g. confidence intervals)
☐	☒	For null hypothesis testing, the test statistic (e.g. *F*, *t*, *r*) with confidence intervals, effect sizes, degrees of freedom and *P* value noted<br>*Give P values as exact values whenever suitable.*
☒	☐	For Bayesian analysis, information on the choice of priors and Markov chain Monte Carlo settings
☒	☐	For hierarchical and complex designs, identification of the appropriate level for tests and full reporting of outcomes
☐	☒	Estimates of effect sizes (e.g. Cohen's *d*, Pearson's *r*), indicating how they were calculated

Our web collection on statistics for biologists contains articles on many of the points above.

Software and code

Policy information about availability of computer code

Data collection	SLiM v3
Data analysis	R v4.0.2; cutadapt v2.3; samtools markdup v1.10; GATK HaplotypeCaller v4.1.0, ART-MountRainier-2016-06-05, bwa mem v2, vcftools 0.1.13, SLiM v3, MACS2, mkTest.rb v1,

For manuscripts utilizing custom algorithms or software that are central to the research but not yet described in published literature, software must be made available to editors and reviewers. We strongly encourage code deposition in a community repository (e.g. GitHub). See the Nature Research guidelines for submitting code & software for further information.

Data

Policy information about availability of data

All manuscripts must include a data availability statement. This statement should provide the following information, where applicable:

- Accession codes, unique identifiers, or web links for publicly available datasets
- A list of figures that have associated raw data
- A description of any restrictions on data availability

Data Availability
Data are available in the Supplementary Tables and Supplementary Data. Mutation data used as our training set are deposited on Figshare (https://doi.org/10.25386/genetics.6456065). Raw Illumina sequencing reads from additional mutation accumulation experiments are available via NCBI SRA. Genomes of natural Arabidopsis thaliana accessions are available at http://1001genomes.org/data/GMI-MPI/releases/v3.1/ . Chromatin state data are available through the Plant Chromatin State Database (http://systemsbiology.cau.edu.cn/chromstates). Raw reads from the ATAC-seq experiments are available via NCBI SRA. Tissue-specific expression data are available at https://www.ebi.ac.uk/arrayexpress/experiments/E-MTAB-7978/. There are no restrictions on the availability of data used

in this study.

Code availability
Annotated scripts of code used in this study will be made available on Github (https://github.com/greymonroe).

Field-specific reporting

Please select the one below that is the best fit for your research. If you are not sure, read the appropriate sections before making your selection.

☒ Life sciences ☐ Behavioural & social sciences ☐ Ecological, evolutionary & environmental sciences

For a reference copy of the document with all sections, see nature.com/documents/nr-reporting-summary-flat.pdf

Life sciences study design

All studies must disclose on these points even when the disclosure is negative.

Sample size	Sample sizes of 107 (original mutation accumulation experiment), 50+ (second mutation accumulation experiment, reflects number of lines per founder genotype), 64 number of individual sequenced leaves to detect somatic mutation) , and 10 (number of siblings of one original mutation accumulation line) Individuals for calling de novo mutations are limited primarily be sequencing costs and the number measured was deemed sufficient as shared variants are expected to be readily identifiable.
Data exclusions	Sequence variants were excluded that failed filtering thresholds based on quantitative estimates of quality or confidence.
Replication	We reproduced our finding that mutation rates are elevated upstream and downstream of transcribed regions with additional sequencing of mutation accumulation line siblings (repeated once). We further replicated our results with analyses of somatic and germline mutation distributions in additional datasets (repeated twice). The first of these was the largest mutation accumulation dataset conducted to date. The second of these was a reanalysis of somatic variants detected in single Arabidopsis leaves. We reproduced the observation of elevated polymorphism rates in 3` and 5` coding regions with analysis of an outgroup species, Populus trichocarpa (repeated once).
Randomization	Mutation accumulation lines were randomized for growing conditions (i.e. position on tray).
Blinding	Investigators were blinded to the location of mutations during variant filtering.

Reporting for specific materials, systems and methods

We require information from authors about some types of materials, experimental systems and methods used in many studies. Here, indicate whether each material, system or method listed is relevant to your study. If you are not sure if a list item applies to your research, read the appropriate section before selecting a response.

Materials & experimental systems

n/a	Involved in the study	
☒	☐	Antibodies
☒	☐	Eukaryotic cell lines
☒	☐	Palaeontology and archaeology
☒	☐	Animals and other organisms
☒	☐	Human research participants
☒	☐	Clinical data
☒	☐	Dual use research of concern

Methods

n/a	Involved in the study	
☒	☐	ChIP-seq
☒	☐	Flow cytometry
☒	☐	MRI-based neuroimaging
